# Selective fetal reduction in monochorionic twins: Preliminary experience

**DOI:** 10.4274/jtgga.galenos.2018.2018.0052

**Published:** 2019-05-28

**Authors:** Vatsla Dadhwal, Aparna K. Sharma, Dipika Deka, Latika Chawla, Nutan Agarwal

**Affiliations:** 1 Department of Obstetrics and Gynecology, All India Institute of Medical Sciences, New Delhi, India

**Keywords:** Monochorionic, selective fetal reduction, bipolar cord coagulation, interstitial laser, radiofrequency ablation

## Abstract

**Objective:**

In complicated mono-chorionic twin pregnancies, vaso-occlusive techniques like bipolar cord coagulation (BPCC), radiofrequency ablation (RFA), interstitial laser ablation (ILA) of cord and fetoscopy guided cord coagulation with lasers are the methods proposed for selective fetal reduction. This study brings forth preliminary data of selective fetal reduction procedures at a tertiary care center in India.

**Material and Methods:**

This was a prospective observational study of 31 patients with complicated mono-chorionic twin pregnancies. Methods used were ILA, RFA and BPCC. Outcome measures included overall co-twin survival after selective feticide, survival rates with each method, miscarriage (defined as all fetal loss before 24 weeks), early fetal death (<24 hours after procedure) and late fetal death (>24 hours after the procedure) of co-twin.

**Results:**

Technical success was achieved in 30/31 (96.8%) of pregnancies. Over all take home baby rate was 63.3%. Live birth rates were 50%, 71.4% and 75% with ILA, RFA and BPCC respectively.

**Conclusion:**

Data from initial cases of selective fetal reduction in complicated mono-chorionic twins suggests that these procedures are feasible but are associated with high adverse perinatal outcome.

## Introduction

It is a well-accepted fact that multiple pregnancies have more maternal complications (abortion, preterm labor, preterm pre-labor rupture of membranes, hypertension in pregnancy, anemia, ante and post-partum hemorrhage, malpresentation, cesarean section) and fetal complications (malformations, intrauterine fetal growth restriction, and complications of prematurity) (1). Therefore, with triplet and higher order gestation, fetal reduction to achieve a total number of two live fetuses is offered to couples with an aim of minimizing these complications. Fetal reduction from twin to singleton in dichorionic twins is debatable, but selective termination in twin gestation discordant for malformations or genetic abnormality is acceptable (2).

In monochorionic twins, fetal reduction may be performed for indications other than twins discordant for anomalies. Monochorionic twins have a unique set of complications such as twin-to-twin transfusion syndrome (TTTS), selective fetal growth restriction, and twin reversed arterial perfusion sequence (TRAP). These complications are due to the presence of inter-fetal vascular anastomoses, which may put one twin at risk of death and adversely affect the health of the other twin. In the event of one twin dying, the transfer of a significant amount of blood from the normal to the dying fetus, through these placental vascular anastomoses, may occur leading to hypotension, hypo-perfusion of the brain leading to cerebral injury (20-30%) and fetal demise (up to 10%) (3,4,5). In a situation where death in one twin is imminent but pregnancy is very preterm, resorting to fetal reduction can optimize outcomes in the surviving twin. Unlike dichorionic pregnancies, fetal reduction using potassium chloride (KCl) instillation in fetal thorax/heart is not an option in mono-chorionic twins due to the presence of placental vascular anastomosis; KCl might transfer to the other fetus and thus inadvertently cause demise of both twins. Vaso-occlusive techniques such as bipolar cord coagulation (BPCC), radiofrequency ablation (RFA), interstitial laser ablation (ILA) of cord, and fetoscopy-guided cord coagulation with laser are the methods proposed for selective fetal reduction in complicated monochorionic twins (6). 

We describe our experience of selective fetal reduction in complicated monochorionic twin pregnancies at a Maternal Fetal Medicine unit in a tertiary care center in India.

## Material and Methods

This is a prospective study that included 31 patients with complicated mono-chorionic twin pregnancies who underwent selective fetal reduction from June 2013 to June 2017, in our unit. The pregnancies were very preterm and at risk of demise of one fetus, which could have adversely affected the other fetus. Informed written consent was obtained from each patient prior to the procedure. The analysis and publication of these data was approved by the institutional ethics committee.

Methods used for cord coagulation were ILA, BPCC, and RFA. ILA was used for fetal reduction in the first half of the study period, whereas in the second half BPCC and RFA was used. The choice of method also depended on the period of gestation and the indication for reduction.

All procedures were performed under ultrasound (US) guidance, using aseptic precautions. Patients received intravenous (i.v.) sedation, injection cefazolin (1 g i.v.) after a sensitivity test, and one dose of 100 mg micronized progesterone intramuscularly (i.m.) prior to the procedure as per the unit protocol. Trocar/needle insertion site was infiltrated with 10 mL of 1% solution of xylocaine.

### Bipolar occlusion of cord

The procedure was performed in pregnancies between 18-26 weeks’ gestation and only if the maximum diameter of the fetal cord was 15 mm or less. Using US guidance, a 3 mm port was inserted into a pocket of amniotic fluid of the affected fetus, preferably at a place away from the placenta. The cord was approached at the abdominal insertion, grasped with the prongs of the bipolar insert, and then pulled to ensure that it was held in its entire width and complete occlusion was confirmed by the absence of flow on color Doppler. Coagulation of the cord was then performed using 20-40 W energy in bursts of 10-15 sec. Echogenic bubbles could be seen and the area of the cord appeared echogenic following the procedure. Two areas of cord were coagulated. The procedure was considered successful if cardiac asystole and absence of flow in the umbilical cord was observed.

### Interstitial laser ablation of cord

The procedure was conducted in pregnancies between 18-26 weeks’ gestation. A 400-micron diode laser was delivered through an 18-G spinal needle under US guidance, targeting intraabdominal fetal umbilical vascular confluence. The laser was fired in short (6-10 sec) pulses at 20-40 W bursts till blood flow ceased.

### Radiofrequency ablation of cord

RFA was performed in pregnancies between 16-27 weeks’ gestation. Under continuous US guidance, a 16-G RFA needle was inserted into the fetal abdomen, alongside the umbilical cord insertion. The prongs of the RFA needle were then deployed in the fetal abdomen and 40 W of energy was delivered to build up the temperature to 100 °C for 2-3 minute till cardiac asystole and cessation of blood flow in umbilical cord was observed; the procedure was repeated in the absence of which. 

In cases with cervical length less than 25 mm, cerclage was performed in the same sitting.

An US examination was performed after 24 hrs to re-document absent cardiac activity in the reduced twin and to check the cardiac activity in the other twin. Middle cerebral artery (MCA) peak systolic velocity (PSV) was measured in the surviving twin to detect fetal anemia, following which the patients were discharged and kept on 2-weekly follow-up. Fetal magnetic resonance imaging (MRI) was performed for some patients after 28 weeks (or 3-4 weeks after procedure) to look for intracranial hemorrhage and cerebral injury. 

Outcome measures included overall co-twin survival after selective feticide, survival rates with each method, miscarriage (defined as all fetal loss before 24 weeks), early fetal death (<24 hours after procedure), and late fetal death (>24 hours after the procedure) of the co-twin.

## Results

Of 31 patients undergoing the procedure, technical success was obtained in 30/31 (96.77%), one patient with a failed procedure was excluded from the analysis. This patient was at 26 weeks with TTTS stage III and gross polyhydramnios, BPCC was unsuccessful. The indications for selective fetal reduction and methods used in the other 30 patients are shown in Table 1.

The mean gestational week at fetal reduction was 23 weeks and 2 days (range, 16-26+4 weeks). Early intrauterine fetal death (IUFD) occurred in 5/30 (16.67%) patients and late IUFD occurred in 1/30 (3.33%); there were 2 spontaneous abortions (6.66%). Both early and late fetal deaths happened in fetuses less than 24 weeks’ gestation. Thus, there were 8 (26.67%) miscarriages (defined as pregnancy loss at or less than 24 weeks gestation).

The mean gestational week at delivery was 35 (range, 26-39) weeks: 13/30 (43.33%) women delivered at or beyond 36 weeks’ gestation, 2/30 (6.67%) delivered between ≥32-36 weeks, 3/30 (10%) delivered at ≥28-32 weeks’ gestation. Of the 4 patients who delivered between 24 and 28 weeks, 3 had stillbirths.

The overall live birth rate was 19/30 (63.3%). There were 3 stillbirths (10%). These three patients delivered between 26-28 weeks’ gestation.

Vaginal birth was achieved in 18/30 (60%) patients. Four babies (21%) required care in the neonatal intensive care unit. The perinatal outcomes of the three procedures are shown in Table 2.

### Follow-up after the procedure

We detected raised MCA PSV after the procedure in 3 cases, one of which aborted subsequently. The other two pregnancies had a normal fetal MRI, the values decreased on follow-up and they delivered a healthy baby at term.

Fetal brain MRI was performed in 14 cases and was found to be normal.

Patients were followed up bi-weekly for growth scans, and monitored for fetal wellbeing. There was no evidence of infection (clinical) in the patients following the procedures.

## Discussion

We evaluated perinatal outcomes after selective feticide in complicated monochorionic twins. The overall survival rate of the co-twin was 63.3%. The survival rate was lower with ILA (50%), whereas survival after RFA and BPCC was similar (71.4 and 75%). A systematic review of selective fetal reduction in 345 complicated mono-chorionic twin pregnancies had an overall fetal survival rate of 79% (65-90%) (2). The authors (2) observed that fetal survival rates were highest with RFA (86%), followed by BPCC (82%), laser cord coagulation (72%), and lowest with cord ligation (70%). The overall survival of the co-twin in our cohort was lower than that reported in this systematic review, though comparable to the study published by Van Den Bos et al. (6) (67.2%). In their series of 131 cases, the survival was lowest with ILA at 46.7%. In our series, the mean gestational age at procedure was higher, this is because of late referrals. Also, TTTS was the indication for reduction in 30% of cases, with most associated with polyhydramnios and short cervix and higher risk of preterm delivery. Procedures with advanced gestation and larger-diameter cords may require more time and multiple cycles of coagulation leading to inter twin transfusion during the procedure, and increasing the risk of demise of the normal twin.

Studies have shown that fetal loss is higher when the procedure is performed before 18 weeks’ gestation (6). As also shown in the systematic review (2), survival rates were better if the procedures were performed after 18 weeks (89% vs 69%). Yinon et al. (7) compared RFA and BPCC and found similar overall survival rates (88.9% vs 76.5%). Selective intrauterine growth restriction as a primary indication for feticide, compared with TTTS, showed a trend towards higher gestational age at delivery and longer procedure to delivery interval. Though the overall survival was similar, the interval between the procedure and delivery was shorter in >24 weeks’ gestation group at the time of the procedure compared with that at <24 weeks. Bebbington et al. (8) compared RFA with BPCC and reported similar success rates. They also reported lower survival if the indication for reduction was TTTS compared with other indications. Sun et al. (9) reported a fetal death rate of 23% after RFA. Variables associated with fetal death were indications for RFA, gestation age >20 weeks, >2 cycles of RFA coagulation, and maximal power setting. In multivariate analysis >2 cycles of RFA coagulation was the only factor independently associated with fetal death (odds ratio: 3.46) (10).

Preterm birth and preterm premature rupture of membranes (PPROM) has also been reported as an important cause of perinatal morbidity and mortality in other series (6,8). Van Den Bos et al. (6) reported an overall PPROM rate of 19.8% with 43.5% babies born between 28 and 37 weeks’ gestation. Bebbington et al. (8) reported an overall PPROM rate of 21.9% after RFA and BCC with preterm delivery (<34 weeks) in 59% of RFA and 44% of BCC procedures (11). In our series, 30% women delivered before 32 weeks.

The procedure is performed to avoid neurologic sequelae in the co-twin when one twin is at risk of death and too premature to deliver. However, we need to understand that survivors after selective feticide in monochorionic twins are at increased risk of neurodevelopmental delay. Van Klink et al. (10) reported neurodevelopmental impairment in 6.8% of surviving twins at a minimum follow-up of 2 years. This is very important and should be part of pre-procedure counseling. We performed antenatal MRI after the procedure in a small number of cases.

Fetal reduction procedures in monochorionic twins are considered to be considerably complex and challenging. They require expertise with hand-eye-needle coordination and a great deal of patience, as needed for most fetal medicine procedures performed under US guidance. Anterior placenta poses technical difficulty because introducing the trocar/needle might result in hemorrhage into the amniotic cavity, which could limit visibility and may also compromise the fetuses. The position of the fetus is also crucial to the success of the procedure. With the fetal spine up towards the maternal abdomen, gaining access to the umbilical cord insertion during ILA/RFA might be difficult. During BPCC, the cord may slip from between the prongs resulting in exsanguination from an incompletely coagulated cord. The presence of poly-hydramnios and short cervical length may contribute to an increased incidence of PPROM and preterm delivery/abortion.

This is probably one of the few studies from developing countries to deal with selective fetal reduction in complicated monochorionic twin pregnancies. The only other studies from our country include a retrospective series of 15 cases of complicated monochorionic twin pregnancies managed by RFA (11) and a single case of complicated TRAP sequence managed by interstitial laser (12). 

Even though we performed the procedure in a small number of patients, this study highlights the feasibility of selective fetal reduction in complicated monochorionic twins in a low-resource setting. The drawbacks are that we could not retrieve the number of women having PPROM from our data, and also the follow-up of babies after discharge was not available. We did not record the cervical length and time/number of cycles required for successful reduction factors known to be risk factors for perinatal outcome. The number of cases of BPCC is small.

Data from initial cases of selective fetal reduction in complicated monochorionic twins suggests that these procedures are feasible but are associated with high adverse perinatal outcomes.

## Figures and Tables

**Table 1:**
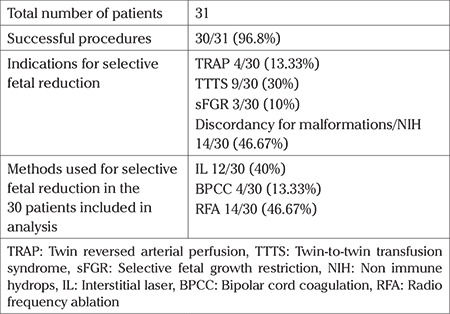
Indications and methods used for fetal reduction

**Table 2:**
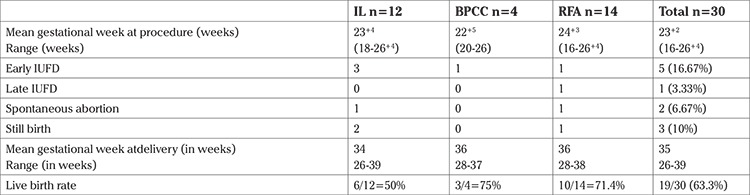
Perinatal outcome according to technique for selective fetal reduction
